# Ultrasound Predictors for Persistence or a Change in the Diagnosis of Rheumatoid Arthritis After 5 Years—A Prospective Cohort Study of Patients with Early Rheumatoid Arthritis

**DOI:** 10.3390/biomedicines13051226

**Published:** 2025-05-19

**Authors:** Tanya Sapundzhieva, Lyubomir Sapundzhiev, Martin Mitev, Rositsa Karalilova, Anastas Batalov

**Affiliations:** 1 Department of Propedeutics of Internal Diseases, Medical Faculty, Medical University of Plovdiv, 4002 Plovdiv, Bulgaria; karalilova@hotmail.com (R.K.); abatalov@hotmail.com (A.B.); 2 Rheumatology Department, University Hospital ‘Pulmed’, 4002 Plovdiv, Bulgaria; sapoundjiev@abv.bg (L.S.); m.mitev97@gmail.com (M.M.); 3 Rheumatology Clinic, University Hospital ‘Sofiamed’, 1113 Sofia, Bulgaria; 4 Rheumatology Clinic, University Hospital ‘Kaspela’, 4000 Plovdiv, Bulgaria

**Keywords:** arthritis, rheumatoid, psoriatic, diagnosis, predictor, ultrasonography, synovitis, tenosynovitis, enthesitis, ultrasound score, biomarker

## Abstract

**Aim:** To identify ultrasound (US) predictors of persistence or change in the diagnosis of rheumatoid arthritis (RA) after five years in a cohort of patients with early RA. **Patients and Methods:** One hundred and twenty patients with early arthritis who met the 2010 ACR/EULAR classification criteria for RA were followed for a period of five years. The US assessment at baseline included a bilateral evaluation of the wrists, second to fifth metacarpophalangeal (MCP) joints, second to fifth proximal interphalangeal (PIP) joints, the IV and VI extensor compartments of the wrists, and the flexor tendons of the second to fifth fingers. This evaluation was conducted using both grayscale ultrasound (GSUS) and power Doppler ultrasound (PDUS). The following scores were recorded for each patient: synovitis score, mini-enthesitis score (including paratenonitis of the finger extensor tendon at the MCP joint, central slip enthesitis at the PIP joint, pseudotenosynovitis, and the A1 pulley of the second finger), finger flexor tenosynovitis score, and tenosynovitis score for the IV and VI wrist extensor compartments. The receiver operating characteristic (ROC) curve was utilized to identify the ultrasound predictors for either maintaining or revising an initial diagnosis of RA. **Results:** At month 6, 82 (68%) patients were classified as having RA according to 1987 ACR RA criteria, 23 (19.2%) were diagnosed with psoriatic arthritis (PsA), 10 (8.3%) with systemic connective tissue disease (SCTD)–8 (6.7%) patients with Sjogren Syndrome and 2 (1.7%) patients with systemic lupus erythematosus (SLE)–and 5 (4.2%) patients with calcium pyrophosphate deposition disease (CPPD). The most significant predictor of RA in the fifth year was the VI extensor compartment tenosynovitis score, with an AUC of 0.915 and a criterion value > 0, associated with a sensitivity of 82.93% and a specificity of 100% (*p* < 0.001). The PDUS synovitis score demonstrated the second-best prognostic ability with an AUC of 0.823, a criterion value > 2, a sensitivity of 82.93%, and a specificity of 73.68% (*p* < 0.001). The mini-enthesitis score showed the best prognostic ability of a PsA diagnosis with an AUC of 0.998, a criterion value > 1, a sensitivity of 95.65%, and a specificity of 100% (*p* < 0.001). The paratenonitis score, pseudotenosynovitis score, and thickened A1 pulley were also predictive of PsA diagnosis with AUCs of 0.977, 0.955, and 0.919, respectively (*p* < 0.001 for all). **Conclusions:** Nearly one-third of the patients who were initially classified as having RA had their diagnosis revised at the end of the fifth year. Ultrasound of joints, tendons, and mini-entheses at baseline may serve as potential imaging predictive biomarkers for persistence or change in diagnosis after 5 years.

## 1. Introduction

Rheumatoid arthritis (RA) is a chronic progressive inflammatory joint disease, affecting up to 1% of adults [1]. The establishment of an early diagnosis, followed by early initiation of effective treatment, and adherence to a treat-to-target strategy, improves the outcomes of the disease in the long term [2,3]. The purpose of the new 2010 American College of Rheumatology (ACR)/European League Against Rheumatism (EULAR) criteria for the classification of RA is to select the patients at risk of having persistent joint inflammation and structural progression, hence to initiate early treatment with disease-modifying antirheumatic drugs (DMARDs), which explains why their sensitivity is high, but specificity is low as compared to the 1987 ACR criteria [4,5,6]. The latter is the reason for misdiagnosing patients, and overtreating them with potentially harmful drugs. Thus, future prospective studies should focus on finding predictive biomarkers for the development of RA, thus increasing specificity and limiting the unnecessary exposure of patients with self-limiting undifferentiated arthritis to DMARDs.

Musculoskeletal ultrasound (MSUS) has been increasingly implemented in everyday rheumatology practice due to the numerous advantages this imaging modality possesses, namely cost-effectiveness, lack of radiation exposure, the possibility of dynamic assessment of multiple areas of interest and patient-friendly nature [7,8].

In 2013, the role of the US in the management of RA patients was highlighted in a set of recommendations, and 3 years later in a set of algorithms—from diagnosis and monitoring of the therapeutic response to assessing the state of remission [9,10]. Thus, the US has been proven to be an imaging biomarker in rheumatology [11,12].

The predictive ability of MSUS has been extensively studied—as a predictor for the establishment of RA diagnosis in patients with arthralgia or with undifferentiated arthritis [13,14,15,16,17,18,19,20,21,22,23,24,25,26,27], as a predictor for remission [12,24,28] and for relapse after drug tapering [29,30,31]. In addition to being a predictive biomarker, the role of the US in distinguishing between early arthritides has been widely evaluated [32,33,34]. Sonographic patterns of the different arthritides have been proven to facilitate the establishment of early diagnosis when clinical phenotype, serological parameters and conventional radiography are inconclusive [32,33,34].

The aim of the current study is to explore whether an extensive US evaluation of joints, tendons and entheses in patients, diagnosed with RA according to the 2010 ACR/EULAR classification criteria, may be predictive of the persistence of the same, or of a change in diagnosis after 5 years.

## 2. Patients and Methods

### Patients

Between February and July 2019, 164 patients with early arthritis, fulfilling 2010 ACR/EULAR criteria for RA [4], were recruited from two centers (University Hospital “Kaspela”, Plovdiv and University Hospital “Pulmed”, Plovdiv) in the study. The study was an open-label, non-randomized. *Inclusion criteria* were as follows: 1. Inflammatory type of joint pain in the small joints of the hands; 2. Symptom duration of less than a year; 3. No erosions on conventional radiography of the hands; 4. No personal or family history of psoriasis; 5. No family history of diseases in the spectrum of spondyloarthropathies (SpA); 6. No prior treatment with disease-modifying antirheumatic drugs (DMARDs) and/or oral corticosteroids; 7. No parenteral corticosteroid (CS) administration (including intra-articular) during the previous six months; 8. Ability to provide written informed consent. *Exclusion criteria* included the following: 1. A prior diagnosis of another rheumatic disease; 2. A history of joint trauma or infection affecting the small joints of the hands; 3. Severe concomitant illnesses could possibly hinder the ability to attend regular visits and complete the 5-year follow-up period.

The patients were followed up for a period of 5 years, including scheduled visits every three months during the first year and every 6 months thereafter if clinical remission was achieved. At baseline, each patient underwent a full physical examination, including a musculoskeletal examination with documentation of the number of tender and painful joints, laboratory tests, and imaging assessment by both conventional radiography and MSUS. All patients included in the current study were presenting with inflammatory arthritis (IA) of the small joints of the hands and had no baseline erosions on an X-ray of the hands.

The study was approved by the Local Ethics Committee of University Hospital ‘Pulmed’ (Protocol №3; Date: 10 January 2019). Prior to inclusion in the study, all patients signed a written informed consent according to the World Medical Association Declaration of Helsinki, revised in 2000, Edinburgh.

## 3. Methods

### 3.1. Clinical Assessment

A single joint assessor (MM) performed the joint evaluation, documenting the number of tender and swollen joints necessary for the calculation of the clinical index for disease activity, known as the disease activity score (DAS) 28, which includes 28 joints: bilateral proximal interphalangeal (PIP), metacarpophalangeal (MCP), wrist, shoulder, elbow, and knee joints [35]. A visual analog scale (VAS) ranging from 0 to 10 centimeters (cm) was used to document the intensity of joint pain, as well as the patient (PtGA) and physician global assessment (PhGA) of the disease. The duration of morning stiffness, joint complaints, and the presence of sicca symptoms (ocular and oral dryness) was also recorded.

### 3.2. Laboratory Assessment

The laboratory parameters tested were full blood count, including differential, kidney (creatinine (44–135 µmol/L), urea (1.7–8.3 mmol/L) and uric acid (148–428 µmol/L) levels, calculation of creatinine clearance in ml/min.) and liver functional tests (aspartate aminotransferase (AST) (0–35 U/L) and alanine aminotransferase (ALT) (0–45 U/L), gamma-glutamyl transferase (GGT) (5–50 U/L)), level of acute-phase reactants like erythrocyte sedimentation rate (ESR) and C-reactive protein (CRP) (reference range 0–5.0 mg/L), urine sediment.

Immunologic tests included measurement of Immunoglobulin M (IgM)-Rheumatoid factor ((IgM-RF: reference range 0–20 U/L), anti-citrullinated protein antibodies (Abs) (ACPA: reference range 0–20 U/L), anti-mutated citrullinated vimentin (MCV) Abs (0–20), antinuclear Abs (ANA) under indirect immunofluorescence (IIF) (negative if <1:80), anti-double-stranded (ds) DNA Abs (0–10), anti-Smith (Sm) Abs (0–10), anti-SS-A Abs (Ro52) (0–10), anti-SS-B Abs (La) (0–10), anti-Scl7 Abs (1–10) and anti-CENP-B Abs (0–10).

### 3.3. Imaging Assessment

#### 3.3.1. Conventional Radiography

Conventional radiography (X-ray) of the hands, feet, and sacroiliac joints was performed at baseline. X-ray of the hands and feet was repeated yearly thereafter for monitoring of the radiographic progression.

#### 3.3.2. Musculoskeletal Ultrasound (MSUS)

##### US Protocol

**Figure 1:**
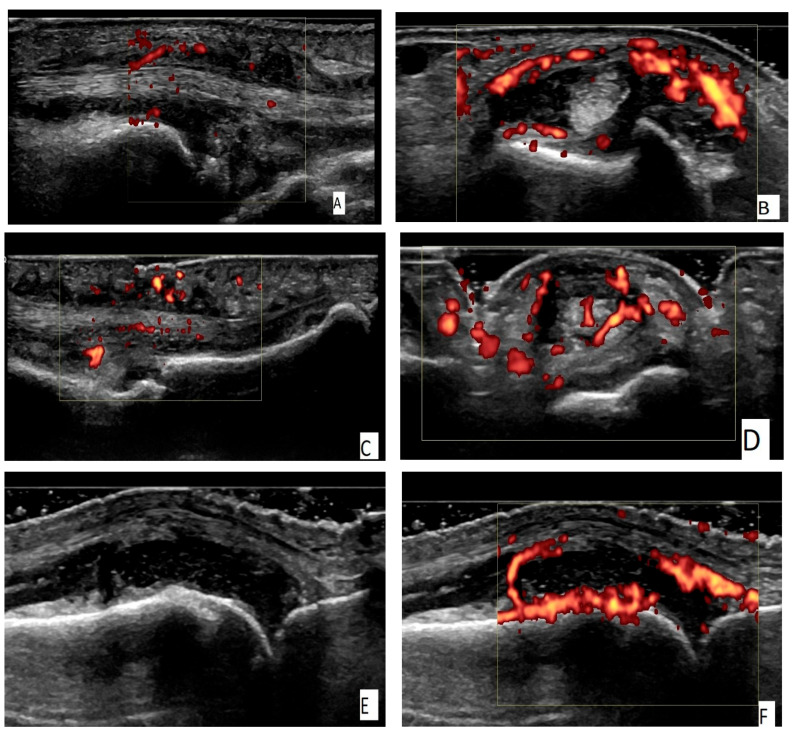

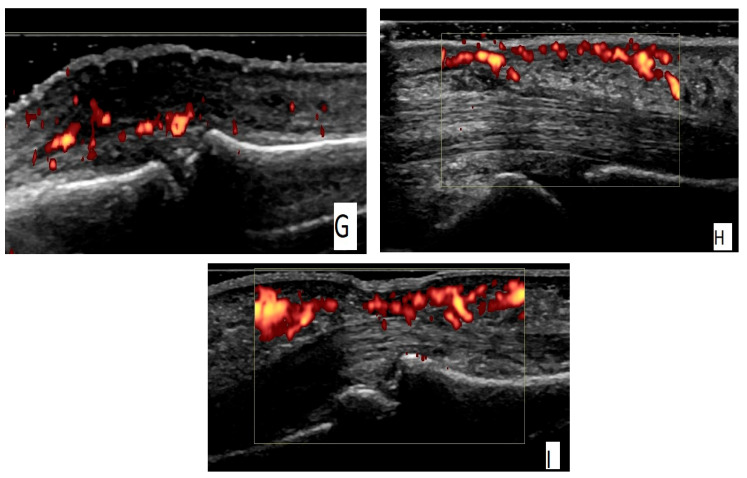
(**A**) An ulnar longitudinal scan of the 6th extensor compartment of the wrist (the tendon of extensor carpi ulnaris—ECU) in an RA patient. Power Doppler positive (PD) tenosynovitis of ECU tendon. (**B**) A transverse scan of the 6th extensor compartment of the wrist (the tendon of extensor carpi ulnaris—ECU) in an RA patient. PD-positive tenosynovitis of ECU tendon. (**C**) A palmar longitudinal scan of an MCP joint in an RA patient. Tenosynovitis of the flexor tendon of the finger with a positive PD signal. (**D**) A palmar transverse scan of an MCP joint in an RA patient. Tenosynovitis of the flexor tendon of the finger with a positive PD signal. (**E**) A dorsal longitudinal scan of an MCP joint in an RA patient. Grade 3 synovitis on a grayscale (GS). (**F**) A dorsal longitudinal scan of an MCP joint in an RA patient. PD synovitis grade 3. (**G**) A dorsal longitudinal scan of a PIP joint in a PsA patient. Paratenonitis of the extensor tendon of the finger with a positive PD signal. (**H**) A palmar longitudinal scan of an MCP joint in a PsA patient. Subcutaneous edema—pseudotenosynovitis of the finger flexor tendon with a positive PD signal. (**I**) A palmar longitudinal scan of a PIP joint in a PsA patient. Pseudotenosynovitis of the finger flexor tendon with a positive PD signal.

Two independent US assessors (TS, RK), who are certified by EULAR and are specialists in MSUS, conducted the US scanning in accordance with the 2017 EULAR recommendations for the performance of standardized US scanning in rheumatology [36]. The wrist (radiocarpal and intercarpal joints), 2nd to 5th MCP and 2nd to 5th PIP joints, flexor and extensor tendons and mini-entheses of the hands were assessed bilaterally by grayscale US (GSUS) and power Doppler US (PDUS). Longitudinal and transverse scans were obtained for all of the scanned structures (Figure 1A–I).

We conducted all ultrasound (US) assessments using the same machine—MyLab 7, Esaote S.p.A., Genova, Italy (equipped with a high-frequency linear probe operating at 6–18 MHz. The physicians performing the US examinations did not have access to the data from the physical examination or laboratory results. The GS frequency was set at 18 MHz, with gain adjustments averaging 50%, depending on the joint examined and patient characteristics, to achieve maximum image resolution. The PDUS frequency was set at 9.1 MHz, with a pulse repetition frequency ranging from 500 to 750 Hz, and low settings for the wall filter.

Dorsal and palmar scans of the radiocarpal and intercarpal joints were obtained to assess the presence of synovitis. A dorsal scan at the wrist joint was performed to assess the IV extensor compartment (including the tendon of extensor digitorum communis) for the presence of tenosynovitis. An ulnar scan was used to evaluate for the presence of tenosynovitis of the VI extensor compartment of the wrist (tendon of extensor carpi ulnaris).

Both dorsal and palmar scans of the MCP joints of the 2nd to 5th fingers were performed to detect synovitis. Only a dorsal scan was used to check for inflammation of the finger extensor tendon, known as paratenonitis of the finger extensor tendon. The palmar scan of the 2nd to 5th MCP joints was utilized to identify inflammation of the flexor tendon, referred to as flexor tenosynovitis, as well as inflammation of the fibrous skeleton above the flexor tendon, indicated by subcutaneous edema with a positive PD signal, also known as ‘pseudotenosynovitis’. The A1 pulley of the flexor tendon was evaluated at the level of the MCP joints on a palmar scan of the second to fifth fingers.

A dorsal scan was performed on the PIP joints of the 2nd to 5th fingers to assess for synovitis and inflammation at the attachment point of the central slip of the extensor tendon at the level of the proximal phalanx, known as central slip enthesitis (CSA). A palmar scan of the PIP joints was conducted to check for inflammation of the finger flexor tendon, also known as finger flexor tenosynovitis.

The recorded pathologies included synovitis of the joints, tenosynovitis of the IV and VI extensor compartments of the wrist, tenosynovitis of the flexor tendons of the fingers, and mini-enthesitis, which encompasses several pathologies—paratenonitis of the extensor tendon at the MCP joints, central slip enthesitis at the level of the PIP joints, pseudotenosynovitis, and A1 pulley inflammation with a positive PD signal.

The presence of synovial hypertrophy, regardless of the presence of joint effusion, was documented as described by the EULAR-OMERACT in 2017 [37,38]. Synovitis by GSUS and PDUS was graded in a semi-quantitative manner from 0 to 2, and synovitis was defined as GS ≥ 2 and/or PD ≥ 1 [37,38].

Tenosynovitis of the IV and VI extensor compartments of the wrist, tenosynovitis of the flexor tendons of the fingers, paratenonitis, central slip enthesitis and pseudotenosynovitis, were graded on a binary scale on both GSUS and on PDUS and given a score of 1 (present) or 0 (absent). The Outcome Measures in Rheumatology (OMERACT) definitions for synovitis, tenosynovitis and enthesitis were used when reporting the pathologic findings [39,40,41].

The A1 pulley was assessed at the MCP joint level as reported by Tinazzi et al. [42]. To define the border between the flexor tendon and the AI pulley, a dynamic US assessment during passive flexion extension of the finger was performed [42]. The A1 pulley was considered inflamed if exhibiting a positive PD signal [42].

Pseudotenosynovitis—defined as edema of the fibrous skeleton surrounding the finger flexor tendon—is characterized by the presence of an abnormal hypoechoic/anechoic area, diffused or localized within the subcutaneous tissue between the epidermis and the tendon-related anatomic structures, accompanied by local thickening, with or without abnormal local Doppler signal, visualized in two perpendicular planes and not evident on the contralateral side [43]. Due to the high variability in the ultrasound features of subcutaneous edema, bilateral comparison is essential. This is particularly important because individual factors—such as age, body mass index (BMI), sex, and hand dominance—can influence the structure and echogenicity of subcutaneous tissue [44]. Paratenonitis of the extensor tendon of the finger is described as a loss of the normal fibrillar echotexture and an increased thickness of the tendon, which exhibits a positive PD signal [45,46].

At the end of the US scanning, six total scores were calculated for each patient. These included the GS and PD synovitis score (a sum of the number of joints with synovitis on GS and PD, respectively), the mini-enthesitis score (a sum of the scores of paratenonitis, central slip enthesitis, pseudotenosynovitis, and A1 pulley inflammation), the IV extensor compartment tenosynovitis score, the VI extensor compartment tenosynovitis score and finger flexor tenosynovitis score. The following scores were recorded for each patient: synovitis score (0–18 points), mini-enthesitis score (0–32), which included assessment of 4 mini-entheses (paratenonitis of the finger extensor tendon at MCP joint, central slip enthesitis at PIP joint, pseudotenosynovitis, and thickened A1 pulley), finger flexor tenosynovitis score of the finger flexors (0–8 points), and tenosynovitis of the IV and VI wrist extensor compartments.

### 3.4. Statistical Methods

The statistical software for the Social Sciences (SPSS) Version 27 (2020) was used to analyze the data. Continuous variables were assessed for normality using the Shapiro-Wilk test. The central tendency was described using means and standard deviations (SD) for normally distributed variables, with between-group comparisons analyzed using independent *t*-tests. Non-normally distributed variables were presented with medians and interquartile ranges (IQR), and between-group comparisons were conducted using the Mann–Whitney test.

Categorical variables were summarized by frequencies and percentages, and associations were determined using the Chi-square test and Fisher’s exact test. The receiver operating characteristic (ROC) curve was employed to evaluate the prognostic ability of clinical and ultrasound (US) markers in maintaining or rejecting an RA diagnosis in the fifth year of follow-up. Spearman rank-order correlation was used to explore relationships between variables when at least one was not normally distributed. All statistical tests were two-tailed and conducted at a Type I error (α) of 0.05.

## 4. Results

### 4.1. Demographic and Clinical Data

The study involved 120 patients who met the 2010 classification criteria of the ACR/EULAR for RA and were followed for five years. By the fifth year, 82 patients fulfilled the 1987 ACR criteria for RA, while the remaining 38 patients had their initial RA diagnosis rejected in favor of other diseases. These included twenty-three patients with psoriatic arthritis (PsA), eight with Sjögren’s syndrome (SS), five with calcium pyrophosphate deposition disease (CPPD), and two with systemic lupus erythematosus (SLE). The patients’ ages ranged from 39 to 63 years, with a mean age of 52.16 ± 5.36 years. The majority of the patients were women (84.20%, *n* = 101). For the study, patients were divided into two groups based on their fifth-year diagnosis: eighty-two patients with unchanged RA diagnosis and thirty-eight patients with a different diagnosis. The demographic data revealed no significant differences between the two groups (see Table 1).

The laboratory parameters showed significant differences between the two groups, as seen in Table 2. Patients with an unchanged RA diagnosis had significantly higher CRP levels (*p* = 0.008). In the RA group, RF showed a higher proportion of patients with high titers (*p* < 0.001). All patients in the group with a changed RA diagnosis tested negative for anti-CCP, while RA patients had either low or high titers (*p* > 0.001). The distribution according to ANA levels also showed significant differences across all levels (*p* < 0.001).

### 4.2. Clinical Predictors of RA Diagnosis on the Fifth Year

ROC curve analysis revealed that anti-CCP was the most reliable predictor of RA diagnosis in the fifth year, with an AUC of 1.00, a criterion value greater than 0 (Negative), a sensitivity of 100%, and a specificity of 100%. Despite its significance, RF cannot be considered a reliable predictor of RA diagnosis in the fifth year, as it had an AUC of 0.691, a sensitivity of 29.27%, and a specificity of 100%. Similarly, CRP was not confirmed as a reliable marker for RA diagnosis in the fifth year, as it showed an AUC of 0.651, a sensitivity of 92.68%, and a specificity of 34.21% (Table 3).

### 4.3. US Predictors of RA Diagnosis in the Fifth Year

Four US indexes at baseline showed significantly higher mean values in the patients with unchanged RA diagnosis in the fifth year, including GSUS synovitis score (*p* = 0.008), PDUS synovitis score (*p* < 0.001), IV extensor compartment tenosynovitis (*p* < 0.001), and VI extensor compartment tenosynovitis (*p* < 0.001). The other six indexes had significantly higher mean values in the patients whose RA diagnosis was changed to a different one in the fifth year. These included the scores for paratenonitis, CSE, pseudotenosynovitis, inflamed A1 pulley, mini-enthesitis, and finger flexor tenosynovitis (*p* < 0.001 for all comparisons) (See Table 4).

The four US indexes with significantly higher mean values in the patients with an unchanged RA diagnosis were evaluated as prognostic markers of RA in the fifth year through ROC curve analysis. The most reliable predictor of RA in the fifth year was the VI extensor compartment tenosynovitis, with an AUC of 0.915 and a criterion value > 0, resulting in a sensitivity of 82.93% and a specificity of 100%. The PDUS synovitis score demonstrated the second-best prognostic ability with an AUC of 0.823, a criterion value > 2, a sensitivity of 82.93%, and a specificity of 73.68%. The IV extensor compartment tenosynovitis and, particularly, GSUS, exhibited relatively low prognostic power, notwithstanding their significance (Table 5).

### 4.4. Predictors of PsA Diagnosis in the Fifth Year

The 23 patients whose initial RA diagnosis was changed to PsA in the fifth year of the follow-up were compared to the group with an unchanged RA diagnosis on the US indexes at baseline. The results showed significantly higher means in the PsA patients for the following scores: paratenonitis, CSE, pseudotenosynovitis, inflamed A1 pulley, mini-enthesitis, and finger flexor tenosynovitis (*p* < 0.001 for all comparisons).

The PDUS synovitis score, the IV extensor compartment tenosynovitis, and the VI extensor compartment tenosynovitis score were significantly higher in the RA group, whereas GSUS did not differ significantly (See Table 6).

Four US indexes demonstrated strong predictive ability for a PsA diagnosis, with an AUC higher than 90%. The mini-enthesitis score showed the best prognostic ability of a PsA diagnosis against an initial RA diagnosis, with an AUC of 0.998, a criterion value > 1, a sensitivity of 95.65% and a specificity of 100%. The paratenonitis score was found to be a reliable predictor of a PsA diagnosis, with an AUC of 0.977, a criterion value > 0, a sensitivity of 96.65%, and a specificity of 98.78%. The pseudotenosynovitis score was also associated with a good prognostic ability with an AUC of 0.955, a criterion > 0, a sensitivity of 91.30% and a specificity of 98.78%. Inflamed A1 pulley had an AUC of 0.919, a sensitivity of 86.96%, and a specificity of 93.90% at a criterion value > 0. The remaining two indexes, finger flexor tenosynovitis and GSE, were found to be less reliable prognostic markers for a PsA diagnosis (Table 7).

## 5. Discussion

It is a well-known fact that early diagnosis and treatment of patients with inflammatory joint diseases are the cornerstones of modern rheumatology. The validation of the 2010 ACR/EULAR criteria for classifying RA patients, due to their high sensitivity, represents a significant step toward the earlier introduction of DMARDs for patients at risk of persistent synovitis. On the other hand, their lower specificity can lead to a not-so-uncommon diagnostic shift and the eventual establishment of an alternative diagnosis during patient follow-up.

The aim of our prospective study was to find what proportion of the early arthritis patients, initially fulfilling the 2010 ACR and EULAR classification criteria for RA, would fulfill the 1987 ACR RA criteria and what proportion would be diagnosed with another rheumatic disease after a period of 5 years. Considering the different therapeutic responses to drugs targeting different cytokines, we set out to explore clinical and imaging predictors of diagnosis persistence and whether specific US features could serve as predictors of a diagnostic shift during the evolution of the inflammatory joint disease.

The choice of the US as the imaging biomarker for predicting the final diagnosis is supported by a substantial body of evidence regarding its role in diagnosis, differential diagnosis, monitoring treatment efficacy, its prognostic significance as an outcome of the disease, as well as its widespread use in daily rheumatology practice largely due to its low cost and safety. The selection of specific joints, tendons, and mini-entheses was guided by current research on the role of ultrasound in distinguishing RA from other inflammatory joint diseases [7,11,32].

The US assessment did not include the metatarsophalangeal (MTP) joints of the feet because there is insufficient data on the role of forefoot ultrasound scanning in differentiating between various types of arthritis. Additionally, the study focused on patients who presented with inflammatory pain specifically in the small joints of the hands. It is also rare for RA patients to show isolated MTP inflammation in the feet without related inflammatory findings in the hands and fingers [27]. Moreover, excluding the feet from the ultrasound protocol helped reduce the overall duration of the US examination.

Many studies have reported the presence of US pathologic findings in healthy individuals [47,48]. All published studies that assessed the predictive ability of US for the progression of preclinical RA—without the presence of synovitis during physical examination— to RA, considered the presence of at least grade two synovitis on GS and any grade of Doppler positivity to be pathological [13,14,15,16,17,18,19,20,21,22]. Therefore, we decided to score synovitis if being at least grade 2 on GS and any grade of PD positivity to be PD-positive synovitis.

Regarding the clinical predictors for the persistence of RA diagnosis, our study has reaffirmed existing knowledge—ACPA positivity is highly specific for RA when compared to other inflammatory joint diseases [49]. ACPA positivity emerged as the most reliable predictor of RA diagnosis in the fifth year, surpassing RF and CRP levels measured at baseline. Despite its significance, RF cannot be deemed a reliable predictor of RA diagnosis in the fifth year. And so, the use of US at baseline is particularly important for those patients who are initially ACPA-negative or have a low positive RF.

In our study, 68.30% of patients retained their RA diagnosis, while 31.70% had their diagnosis changed by the fifth year. The distribution of patients’ sex was proportional between the two groups. The patients who maintained the same RA diagnosis in the fifth year exhibited significantly higher mean values in four ultrasound (US) indexes, which included baseline GSUS and PDUS synovitis scores, as well as IV and VI extensor compartment tenosynovitis. Among these, the most reliable predictor of RA in the fifth year was VI extensor compartment tenosynovitis, while the PDUS synovitis score showed the second-best prognostic capability. IV extensor compartment tenosynovitis and, notably, GSUS displayed relatively low prognostic power, despite their significance.

Filer et al. followed 58 patients with at least one joint presenting with clinical synovitis for e period of 18 months and found that GS synovitis of the wrist and MCP joints and PD synovitis of the MTP joints were predictive of meeting the 1987 ACR and the 2010 ACR/EULAR criteria for RA [15]. In a study from 2018, tenosynovitis of the flexor tendons of the fingers had been proven to have a higher predictive value than ACPA and US-detected synovitis for the development of persistent RA in early arthritis patients [50]. Filippucci et al. found that most RA patients showed US evidence of tenosynovitis of extensor carpi ulnaris and the flexor tendons of 2–4 fingers [51]. A systematic literature review confirms that the most commonly affected tendon in RA is in the VI extensor compartment of the wrist [32].

In our study, the patients whose initial diagnosis of RA was changed to PsA during the fifth year of follow-up exhibited significantly higher mean scores in paratenonitis, CSE, pseudotenosynovitis, inflamed A1 pulley, mini-enthesitis, and finger flexor tenosynovitis assessments.

Four US indexes demonstrated strong predictive ability for a PsA diagnosis, with an AUC higher than 90%, including the mini-enthesitis score, the paratenonitis score, the pseudotenosynovitis score, and the score for inflamed AI pulley. The mini-enthesitis score showed the best prognostic ability of a PsA diagnosis against an initial RA diagnosis. The remaining two indexes, finger flexor tenosynovitis and GSE, were found to be less reliable prognostic markers for a PsA diagnosis.

A systematic literature review, published in 2020, found that inflammation of the small entheses of the hands is typical of PsA. Paratenonitis of the extensor tendon at the level of the MCP joints, inflammation of the central slip of the extensor tendon at the proximal phalanx, inflammation of the flexor pulleys of the fingers—especially the A1 pulley—and inflammation of the fibrous skeleton of the fingers, known as pseudotenosynovitis, are highly specific markers for patients with psoriatic arthritis (PsA) when compared to those with RA [32]. Therefore, the presence of these conditions in patients with early arthritis may indicate a diagnosis of PsA rather than RA.

In a prospective 2-year study, Daskareh et al. (2024) found that the thickness of the synovial tissue in the radiocarpal joint, wrist joint effusion, and MCP/PIP joint synovitis predict which patients with inflammatory joint pain and no obvious synovitis during physical examination will develop full-blown RA after 2 years [13].

A study by Mankia et al. compared the US features of patients with palindromic rheumatism (PR) with those of patients with new-onset RA (NORA). As much as 74% of ACPA-positive patients with PR did not develop IA during the follow-up period. Nevertheless, many of them fulfilled the 2010 ACR/EULAR criteria for RA, which may have initiated unnecessary treatment for them. The findings indicate that PR is characterized by extracapsular inflammation—specifically, periarticular inflammation, peritendinous edema, and subcutaneous edema—detected by the US, compared to synovitis and tenosynovitis, which are the most common lesions identified in NORA through US imaging [52].

Two studies have provided evidence that ultrasound (US) increased the sensitivity of the 2010 ACR/EULAR criteria for RA [53,54]. Ji et al. conducted a follow-up study on 94 ACPA-negative RA patients who met the 2010 ACR/EULAR criteria for RA and found that 65 of them received an alternate diagnosis by the end of the first year. PD-positive synovitis of the joints, particularly in the wrists, was identified as the only ultrasound predictor of the persistence of an RA diagnosis [54].

### Limitations

One limitation of our study is that the US was the only assessment modality used to evaluate joint and tendon pathology, without another comparative imaging technique such as MRI. Additionally, the relatively small sample size poses another limitation. Furthermore, the evaluation focused solely on the tendons and joints of the hands, thereby excluding the significant enthesis of the lower limbs and metatarsophalangeal (MTP) joints, which are also frequently affected by inflammatory joint disease.

Our study is the first and only one to examine the predictive ability of the US at baseline for maintaining or rejecting an initial diagnosis after five years. A key strength of the study is its prospective design and the consistent use of the same ultrasound machine throughout the research.

## 6. Conclusions

Nearly one-third of the patients who were initially classified as having RA had their diagnosis revised at the end of the fifth year. Ultrasound of joints, tendons, and mini-entheses at baseline may serve as potential imaging predictive biomarkers for persistence or change in diagnosis after five years.

## Figures and Tables

**Table 1:** Demographic and clinical data.

Variables	RA Diagnosis Unchanged (*n* = 82)	RA Diagnosis Changed (*n* = 38)	*p*-Value
Age ○ Mean (SD) ○ Minimum-Maximum	52.20 (4.88) 41–62	52.07 (6.35) 39–63	0.904 ^t^
Sex n (%) ○ Male ○ Female	12 (14.60%) 70 (85.40%)	7 (18.40%) 31 (81.60%)	0.600 ^f^
Symptom duration (months) ○ Median (IQR)	5.00 (2.00)	5.50 (3.00)	0.260 ^U^
Type of treatment n (%)			
○ MTX ○ LEFL ○ MTX + SSZ ○ HCQ + SSZ	63 (76.80%) 18 (22.00%) 1 (1.20%) 0 (0.00%)	30 (78.90%) 6 (15.80%) 0 (0.00%) 2 (5.30%)	0.152 ^χ2^
Dry eyes n (%)	22 (26.80%)	8 (21.10%)	0.651 ^f^
Dry mouth n (%)	18 (22.00%)	7 (18.40%)	0.810 ^f^
TJC ○ Median (IQR)	8.50 (3.00)	10.00 (4.00)	0.055 ^U^
SJC ○ Median (IQR)	7.00 (2.25)	6.00 (3.25)	0.260 ^U^
VAS (0 to 100) ○ Median (IQR)	70.00 (20.00)	70.00 (10.00)	0.793 ^U^
PtGA (0 to 100) ○ Median (IQR)	70.00 (20.00)	70.00 (10.00)	0.793 ^U^
Morning stiffness (MS) duration ○ <1 h ○ 1 to 2 h	37 (45.10%) 45 (54.90%)	13 (34.20%) 25 (65.80%)	0.321 ^f^

MTX—Methotrexate; LEF—Leflunomide; SSZ—Sulfasalazine; HCQ–Hydroxychloroquine; TJC—Tender joint count; SJC—Swollen joint count; VAS—Visual-analog scale; PtGA—Patient Global Assessment; ^t^—independent samples *t*-test; ^f^—Fisher’s exact test; ^U^—Mann–Whitney U; ^χ2^—Chi-square.

**Table 2:** Laboratory data.

	RA Diagnosis Unchanged (*n* = 82)	RA Diagnosis Changed (*n* = 38)	*p*-Value
CRP (mg/L) ○ Median (IQR)	13.85 (8.15)	9.70 (8.25)	0.008 ^U^
RF (IU/mL) ○ Negative ○ Low titer ○ High titer	11 (13.40%) ^a^ 47 (57.30%) ^a^ 24 (29.30%) ^a^	12 (31.60%) ^b^ 26 (68.40%) ^a^ 0 (0.00%) ^b^	<0.001 ^χ2^
anti-CCP (EU/mL) ○ Negative ○ Low titer ○ High titer	0 (0.00%) ^a^ 59 (72.00%) ^a^ 23 (28.00%) ^a^	38 (100%) ^b^ 0 (0.00%) ^b^ 0 (0.00%) ^b^	<0.001 ^χ2^
ANA ○ Negative ○ 1:80 ○ 1:160 ○ 1:320	6 (7.30%) ^a^ 45 (54.90%) ^a^ 31 (37.80%) ^a^ 0 (0.00%) ^a^	12 (31.60%) ^b^ 12 (31.60%) ^b^ 11 (28.90%) ^b^ 3 (7.90%) ^b^	<0.001 ^χ2^
DAS 28 ○ Moderate activity (>3.2 to 5.1) ○ High activity (>5.1)	29 (35.40%) ^a^ 53 (64.60%) ^a^	17 (44.70%) ^a^ 21 (55.30%) ^a^	0.420 ^f^

CRP—C-reactive Protein; RF—Rheumatoid factor; anti-CCP–Anti-cyclic citrullinated peptide antibodies; ANA—Antinuclear antibodies; ^U^—Mann–Whitney U; ^χ2^—Chi-square; ^f^—Fisher’s exact test; Each superscript letter denotes a subset of the group variable whose proportions do not differ significantly from each other at the 0.05 level.

**Table 3:** Results from ROC curve analysis for clinical predictors of RA diagnosis in the 5th year.

Predictors	AUC	95% CI	*p*-Value	Criterion Value	Sensitivity	Specificity
anti-CCP	1.00	0.970 to 1.00	<0.001	>0 (Negative)	100%	100%
RF	0.691	0.600 to 0.772	<0.001	>1 (Low titer)	29.27%	100%
CRP	0.651	0.559 to 0.736	0.009	>7.50 mg/L	92.68%	34.21%

**Table 4:** Comparison of US indexes at baseline between RA diagnosis unchanged and RA diagnosis changed in the 5th year.

US Indexes	RA Diagnosis Unchanged (*n* = 82)	RA Diagnosis Changed (*n* = 38)	*t*-Test *p*-Value
GSUS synovitis score ○ Mean (SD)	9.93 (2.43)	8.55 (3.01)	0.008
PDUS synovitis score ○ Mean (SD)	3.82 (1.64)	1.76 (1.53)	<0.001
Paratenonitis score ○ Mean (SD)	0.012 (0.11)	1.55 (1.51)	<0.001
CSE score ○ Mean (SD)	0.00 (0.00)	0.34 (0.66)	<0.001
Pseudotenosynovitis score ○ Mean (SD)	0.012 (0.11)	1.42 (1.34)	<0.001
Inflamed A1 pulley ○ Mean (SD)	0.06 (0.24)	0.94 (1.13)	<0.001
Mini-enthesitis score ○ Mean (SD)	0.09 (0.28)	4.26 (3.88)	<0.001
IV extensor compartment tenosynovitis ○ Mean (SD)	0.60 (0.68)	0.02 (0.16)	<0.001
VI extensor compartment tenosynovitis ○ Mean (SD)	1.46 (0.77)	0.00 (0.00)	<0.001
Finger flexor tenosynovitis ○ Mean (SD)	0.91 (1.29)	2.31 (2.05)	<0.001

**Table 5:** Results from ROC curve analysis for US predictors of RA diagnosis in the 5th year.

Predictors	AUC (95% CI)	*p*-Value	Criterion Value	Sensitivity	Specificity
GSUS synovitis score	0.660 (0.568 to 0.744)	0.005	>7	87.80%	42.11%
PDUS synovitis score	0.823 (0.742 to 0.886)	<0.001	>2	82.93%	73.68%
IV extensor compartment tenosynovitis	0.738 (0.650 to 0.814)	<0.001	>0	50.00%	97.37%
VI extensor compartment tenosynovit	0.915 (0.850 to 0.958)	<0.001	>0	82.93%	100%

**Table 6:** Comparison of US indexes between RA and PsA patients in the 5th year.

US Indexes	RA Diagnosis (*n* = 82)	PsA Diagnosis (*n* = 23)	*t*-Test *p*-Value
GSUS synovitis score ○ Mean (SD)	9.93 (2.43)	9.73 (2.87)	0.739
PDUS synovitis score ○ Mean (SD)	3.82 (1.64)	2.00 (1.41)	<0.001
Paratenonitis score ○ Mean (SD)	0.012 (0.11)	2.47 (1.23)	<0.001
CSE score ○ Mean (SD)	0.00 (0.00)	0.56 (0.78)	<0.001
Pseudotenosynovitis score ○ Mean (SD)	0.012 (0.11)	2.04 (1.10)	<0.001
Inflamed A1 pulley ○ Mean (SD)	0.06 (0.24)	1.56 (1.07)	<0.001
Mini-enthesitis Score ○ Mean (SD)	0.09 (0.28)	6.65 (3.02)	<0.001
IV extensor compartment tenosynovitis ○ Mean (SD)	0.60 (0.68)	0.043 (0.20)	<0.001
VI extensor compartment tenosynovitis ○ Mean (SD)	1.46 (0.77)	0.00 (0.00)	<0.001
Finger flexor tenosynovitis ○ Mean (SD)	0.91 (1.29)	3.08 (1.80)	<0.001

**Table 7:** Results from ROC curve analysis for US predictors of PsA diagnosis in the 5th year.

Predictors	AUC (95% CI)	*p*-Value	Criterion Value	Sensitivity	Specificity
Paratenonitis score	0.977 (0.927 to 0.996)	<0.001	>0	95.65%	98.78%
CSE score	0.696 (0.598 to 0.782)	0.002	>0	39.13%	100%
Pseudotenosynovitis score	0.955 (0.895 to 0.086)	<0.001	>0	91.30%	98.78%
Inflamed A1 pulley	0.919 (0.849 to 0.963)	<0.001	>0	86.96%	93.90%
Mini-enthesitis score	0.998 (0.961 to 1.00)	<0.001	>1	95.65%	100%
Finger flexor tenosynovitis	0.835 (0.747 to 0.899)	<0.001	>1	82.61%	76.83%

## Data Availability

Raw data were generated at University Hospital “Pulmed”. Derived data supporting the findings of the study are available from the corresponding author (TS), on request.
